# Toll-Like Receptors Recognize Intestinal Microbes in Liver Cirrhosis

**DOI:** 10.3389/fimmu.2021.608498

**Published:** 2021-02-23

**Authors:** Yujing Fan, Yunpeng Li, Yanjie Chu, Jing Liu, Lin Cui, Dekai Zhang

**Affiliations:** ^1^ Department of Gastroenterology and Hepatology, The Second Affiliated Hospital of Harbin Medical University, Harbin, China; ^2^ Center for Infectious and Inflammatory Diseases, Texas A&M University, Houston, TX, United States

**Keywords:** liver cirrhosis, dysbiosis, gut-liver axis, bacterial translocation, toll-like receptors

## Abstract

Liver cirrhosis is one major cause of mortality in the clinic, and treatment of this disease is an arduous task. The scenario will be even getting worse with increasing alcohol consumption and obesity in the current lifestyle. To date, we have no medicines to cure cirrhosis. Although many etiologies are associated with cirrhosis, abnormal intestinal microbe flora (termed dysbiosis) is a common feature in cirrhosis regardless of the causes. Toll-like receptors (TLRs), one evolutional conserved family of pattern recognition receptors in the innate immune systems, play a central role in maintaining the homeostasis of intestinal microbiota and inducing immune responses by recognizing both commensal and pathogenic microbes. Remarkably, recent studies found that correction of intestinal flora imbalance could change the progress of liver cirrhosis. Therefore, correction of intestinal dysbiosis and targeting TLRs can provide novel and promising strategies in the treatment of liver cirrhosis. Here we summarize the recent advances in the related topics. Investigating the relationship among innate immunity TLRs, intestinal flora disorders, and liver cirrhosis and exploring the underlying regulatory mechanisms will assuredly have a bright future for both basic and clinical research.

## Introduction

Cirrhosis is one of the leading causes of death in clinics among all digestive diseases (1). Cirrhosis refers to a process of a diffuse, progressive, fibrosing, nodular condition in liver tissue, and is a leading cause of chronic liver failure. Many different causes can induce liver cirrhosis (2–11). Cirrhosis progression can be divided into the compensatory stage and decompensated stage. As a result of the late stage, serious complications will occur, and death can hardly be avoided. There are no cure medicines to treat decompensated cirrhosis except for liver transplantation, which needs a rarely available donor liver. Although it is well known that viruses, alcohol, and non-alcoholic steatohepatitis (NASH) are closely associated with the development of cirrhosis, the exact molecular mechanism is still poorly understood. But, regardless of any causes, recent studies have shown that one common feature in cirrhosis is the alteration of gut microbiota, or dysbiosis, which is worsened with the severity of cirrhosis (12).

Intestinal microbes include bacteria, viruses, fungi, parasites, and archaea. In which bacteria are well studied and perhaps play a central role among them (13). In recent years, studies have been found that the correction of altered intestinal flora can delay or maybe revert the progress of cirrhosis (14), and toll-like receptors recognize altered gut microbes to modulate the occurrence, development, and treatment of liver cirrhosis (15).

TLRs are one kind of critical innate immunity pattern recognized receptors (PRRs) that recognize not only pathogen/microbe-associated molecular patterns (PAMPs or MAMPs) but also endogenous damage-associated molecular pattern molecules (DAMPs) (16, 17). Among the TLR family, some TLRs recognize different patterns in bacteria, including TLR1/2 or TLR2/6, TLR4, TLR5, and TLR9, recognizing bacterial lipoproteins, LPS, flagellin, and CpG DNA, respectively (18). TLRs recognize altered microbe in cirrhosis to activate TLR signaling pathway. TLR activation appears to be a critical molecular mechanism and is strongly associated with the progression of the diseases. Therefore, the investigation of the interaction between TLRs and the altered microbe in cirrhosis represents a promising strategy for developing an effective treatment against the deadly cirrhosis. Here we discuss the current understanding of the connection and research advances among dysbiosis, TLR recognition, and cirrhosis.

## Altered Gut Microbiota in Liver Cirrhosis

**Figure 1:**
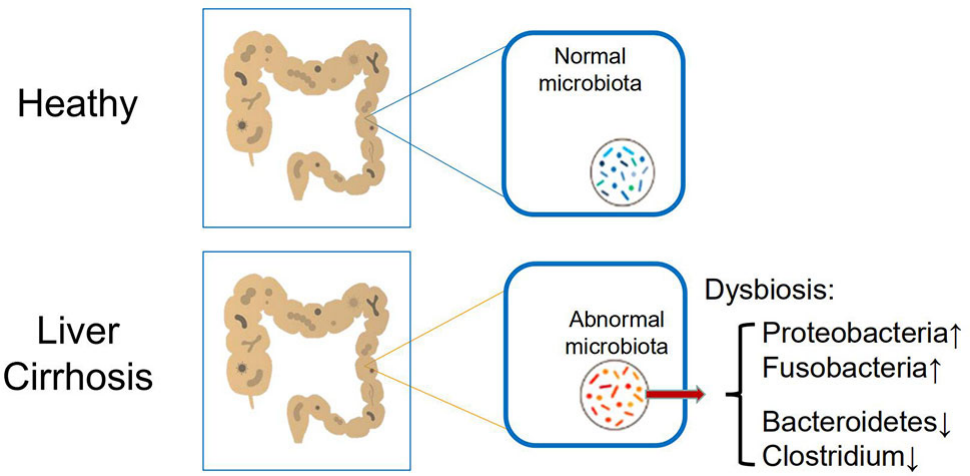
Main alteration of intestinal microbiome in patients with cirrhosis, including increased *Proteobacteria* and *Fusobacteria*, and reduced *Bacteroidetes* and *Clostridium*.

The human gastrointestinal (GI) tract, with about 200–300 m^2^ mucosal surface, is continuously exposed to not only food but also microorganisms. For a healthy person, there are about trillions of microorganisms in the intestine. The gut microorganisms include not only bacteria, but also fungi and viruses, archaea, and parasites. The composition of the human intestinal flora is variable among different individuals and is influenced by many factors, including genetic variation, diet, alcohol consumption, aging, disease status, and whether antibiotics are applied (19, 20). However, the total of quantities and qualities is relatively stable in healthy people. In addition to performing their respective biological functions, the intestinal flora constantly interacts with the host; these interactions affect physiological, immunological, and pathological processes in a variety of cells and tissues (21, 22). One of the recent remarkable discoveries in cirrhosis patients, regardless of causes, is the considerable alteration of microbiota composition in the gut (called dysbiosis) (**Figure 1**) (23, 24).

Although a vast number and species of microorganisms colonize in the human intestines, the healthy gut microbiome is dominated by only some bacterial species, and the quantitative and qualitative changes in cirrhosis, including increased *Proteobacteria* and *Fusobacteria* and reduced *Bacteroidetes* (24). Meanwhile, it has been proved by the analysis of fecal bacterial content that the intestinal flora of the animal liver cirrhosis model and the human liver cirrhosis model will have a significant reduction in microbial diversity compared with the normal human intestinal flora (25, 26). Besides, a reduction in *Clostridium* resulted in significant pro-inflammatory symptoms and was inversely correlated with the Child-Pugh score (27). Furthermore, the specific correlation of mucosal-associated flora changes has been confirmed by significant differences between patients with cirrhosis and patients with hepatic encephalopathy (see below).

How to understand the altered intestinal microbes in liver cirrhosis? Currently, it is still a topic of debate about which comes first between the changing gut microbiota and liver cirrhosis. Liver cirrhosis may induce the alteration of microbes in the intestine, but we assume that the gut microbiota may change first. The alteration of gut microbiota is then recognized by innate immunity receptors such as Toll-like receptors to induce a serial cascade of immune responses and to gradually affect liver development of cirrhosis.

## Toll-Like Receptors Recognized Intestinal Microbiota in Liver Cirrhosis

One of the most exciting and revolutionary discoveries in modern life science and medicine is to realize how important our innate immunity to human health and diseases. In 1989, Charles Janeway first proposed a concept of the Pattern Recognition Receptor (PRR), which recognizes pathogen-associated molecular patterns (PAMPs) to activate not only innate immunity but also adaptive immunity (28). Driven by this hypothesis, the Toll-like receptor (TLR) is the first identified PRR. TLRs are a type-I transmembrane protein capable of sensing pathogen infection. PRRs not only include Toll-like receptors (TLRs) but also include Nucleotide-binding oligomerization domain-like receptors (NLRs), C-type lectin receptors (CLRs), and Retinoic acid-inducible gene I-like receptors (RLRs). Among these PRRs, TLRs are the main receptors that recognize intestinal bacteria. It has been reported that TLRs are very important in the occurrence, development, and treatment of liver cirrhosis, but the most important role of TLRs in cirrhosis is perhaps to identify intestinal flora to trigger signaling cascades which link to the progress of liver cirrhosis.

**Figure 2:**
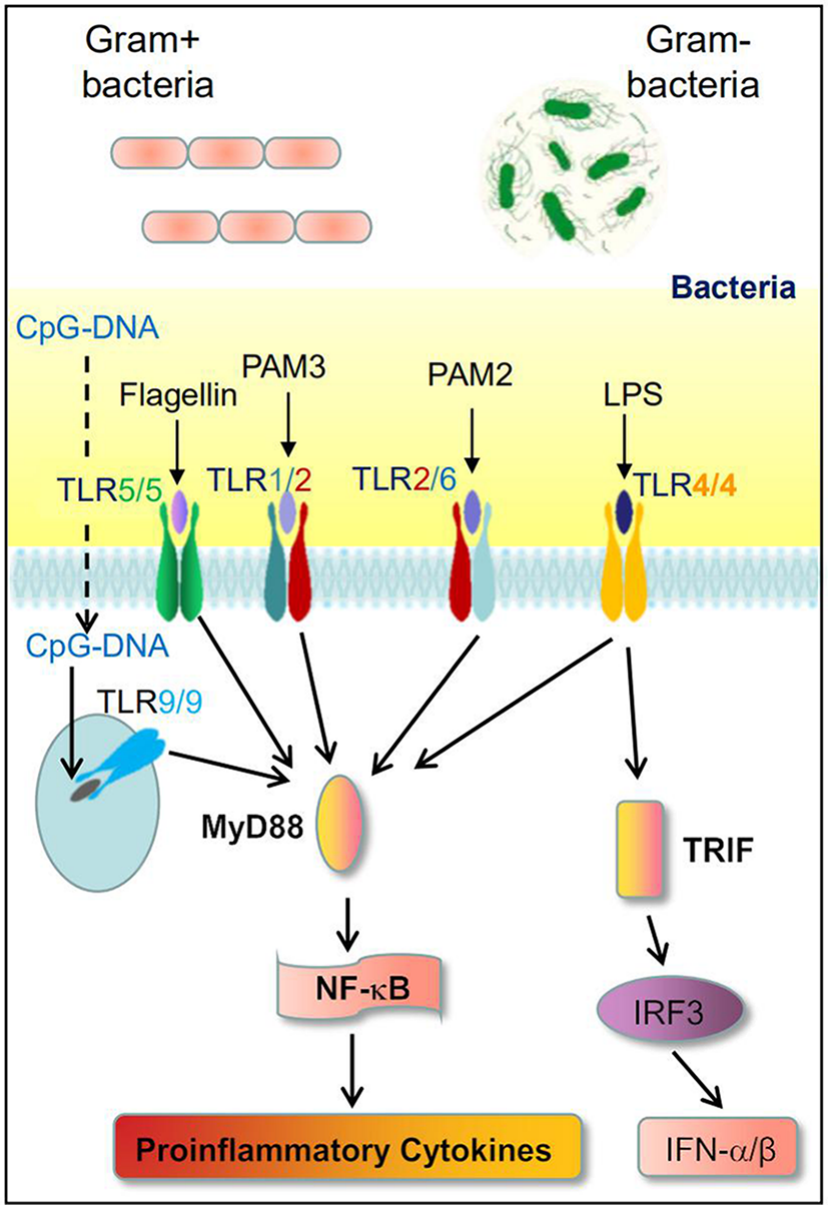
TLRs recognize gut bacteria to induce immune response.

A TLR is the key element of the innate immune system. Among 10 human TLRs, TLR2, TLR4, TLR5, and TLR9 recognize bacterial infection (**Figure 2**). TLR4 has been the most studied. Lipopolysaccharide (LPS) is a bacterial wall component from Gram-negative bacteria and is a common natural ligand of TLR4. TLR4-LPS association requires LPS binding protein, CD14, and myeloid differentiation protein 2 (MD2) to recognize LPS (29). TLR4 activated cell signal transduction has been well confirmed, mainly involving bone marrow differentiation primary response 88 (MyD88) dependent and MyD88 independent. Dependent signal transduction, which promotes mitogen-activated protein kinase and JNK aggregation to produce inflammatory activation through nuclear factor (NF-κB) (30). Cell death induced by these pathways further stimulates the recruitment of neutrophils and other inflammatory cells to the liver (31). Intestinal flora affects intestinal permeability and increases endotoxin load in portal vein circulation (32). This endotoxemia level does not produce septicemia syndrome, but it can produce a systemic pro-inflammatory and fibrotic environment, in which insulin signal transduction is damaged, resulting in increased net fat decomposition in adipose tissue and the transport of free fatty acids (FFA) from adipose tissue to the liver (33). Once excessive lipid is exposed to cellular stress in the liver, it is further expanded through intrahepatic and systemic proinflammatory environments. After that, it showed an acute reaction, hepatic fibrosis, and further progress into liver cirrhosis (34). In contrast to TLR4 recognizing Gram-negative bacteria, TLR2, heterodimer with TLR1 or TLR6, mainly recognizes Gram-positive bacteria. TLR5 and TLR9 recognize both Gram-positive and Gram-negative bacterial flagellin and CpG DNA, respectively.

A lot of evidence has shown that the intestinal flora may play a major role in the pathogenesis of cirrhosis. As mentioned above, we are still not sure which one occurs first between the altered intestinal microbiome and liver cirrhosis. Next, we will discuss the potential mechanism underlying the dysbiosis in cirrhosis.

## Understanding the Link Between Intestinal Microbiota and Liver Cirrhosis

As to how the intestinal flora causes cirrhosis, the following mechanisms have been proposed. The first mechanism mainly involves Toll-like receptors. As discussed above, intestinal flora in cirrhotic patients changes significantly, thus breaking the normal balance. It is still difficult to determine whether the intestinal flora changes before cirrhosis, or whether cirrhosis occurs first and then intestinal flora imbalance occurs. However, it can be determined that the imbalance of intestinal flora will cause inflammation, which can lead to and aggravate liver fibrosis. Intestinal flora imbalance triggers inflammation by activating PRR (35). The most common class of PRR is Toll-like receptors. The liver has strong innate immunity in the human body, and many TLRs are expressed and can be activated in the liver (36), which will not be discussed in detail here. Briefly, multiple TLRs play a critical role in the liver in monitoring and eliminating the invasion of pathogens under normal physiological conditions. While certain microbial-derived molecules can be tolerated for the monitoring and elimination of the liver, the liver cells may produce an immune response against these potentially harmful stimuli, thereby producing an immune response (37). Furthermore, TLRs have been found in many types of hepatocytes, including the bile duct epithelium, the dendritic cells, the endothelial cells, the stellate cells, Kupffer cells, and hepatocytes (38).

The second mechanism involves bacterial translocation. Because of abnormal immune defense, cirrhotic patients are prone to bacterial infection, especially those with advanced liver disease (Child-Pugh B and C). The Child-Pugh score consists of five clinical features and is used to assess the prognosis of liver cirrhosis, in which Child-Pugh B: 7–9 points (significant functional compromise); Child-Pugh C: 10–15 points (decompensated disease). The main causes of infection in patients with liver cirrhosis may be the decrease of phagocytic activity of bacterial translocation, reticular endothelial system from intestinal to mesenteric lymph nodes, and the decrease of antibacterial activity of ascitic fluid (39). The current pathogenic mechanism to explain the passage of bacteria or their products from the intestinal lumen through the intestinal barrier and to mesenteric lymph nodes is defined as bacterial translocation (BT). Generally proved by positive bacterial culture in mesenteric lymph nodes (40). BT also was found in patients with liver cirrhosis who underwent laparotomy (41). Patients with advanced liver disease occur more frequently. Besides, in recent years, it has been shown that BT in patients with liver cirrhosis is related to factors other than bacterial infection, such as coagulation, renal dysfunction, and multiple organ failure (42).

The changes of BT in patients with liver cirrhosis may be due to multiple reasons including intestinal bacterial overgrowth (IBO), intestinal barrier damage, and local immune defense. It has been observed in the experimental model of liver cirrhosis and reaction of the bacterial overgrowth in patients with cirrhosis of the liver, the main growth of bacteria is aerobic Gram-negative bacteria (25), which is closely related to the development of BT and Spontaneous bacterial peritonitis (SBP). Oral antibiotics, which can inhibit Gram-negative aerobic gut bacteria, can significantly reduce the incidence of SBP in patients with cirrhosis and liver cirrhosis models of IBO, BT, and the incidence of bacterial peritonitis (43).

The most important way to prevent IBO in the small intestine is to maintain normal gastric secretion, normal intestinal movement, and ileocecal valve integrity. Inhibition of gastric acid secretion is associated with IBO, especially in the elderly (44). However, recent data show that short-term treatment of neonatal rats with H2 receptor antagonists is not conducive to the reduction of BT (45).

Decompensated cirrhotic patients often receive long-term treatment with anti-H2 receptor antagonists, which can prevent gastrointestinal bleeding caused by acute erosion of gastric mucosa or esophageal scar bleeding after endoscopic treatment of esophageal varices. Recent data show that patients with liver cirrhosis treated with gastric acid secretion inhibitors for a long time have a higher incidence of IBO, especially in patients with advanced liver disease (42). A recent retrospective study showed an increase in the incidence of SBP in patients treated with proton pump inhibitors for gastric acid inhibition (45).

## Mechanisms Underlying the Dysbiosis in Cirrhosis

What is the underlying mechanism underlying the dysbiosis in cirrhosis? There are no clear answers yet, but there are some different pieces of evidence to support the different approaches for the alterations of microbe in the gut of cirrhosis patients. One of the resources of these bacteria mainly came from oral. Thirteen species of microbe in cirrhosis gut were closest to oral isolates. It is concluded that oral flora may invade the intestines of patients with liver cirrhosis and cause intestinal flora changes (46). Another possible mechanism is because of the bile resistance of human intestinal bacteria, the decrease of bile production in patients with liver cirrhosis makes the intestine more likely to be allowed or close to by “foreign” bacteria (47). Some experiments have also found strains such as *Campylobacter jejunum* and parainfluenza in the intestinal flora of patients. Pathogens such as *Hemophilus*, which may invade the intestines through oral pathways or contaminated food. Invasive foreign bacteria can grow not only in the colon, but also in the ileum, and contribute to the excessive growth of bacteria in the small intestine associated with liver cirrhosis, thus aggravating the development of liver cirrhosis (48).

The newly identified mechanism is Small intestinal bacterial overgrowth (SIBO). SIBO is defined as the number of bacteria in the small intestine >10^5^ CFU/ml or the presence of colon bacteria in the upper Jejunum secretions. According to this standard, the experiment found that the incidence of SIBO in patients with liver cirrhosis was between 48 and 73% (49, 50). SIBO is particularly common in patients with severe liver cirrhosis and patients with spontaneous peritonitis or a history of hepatic brain disease. In the late stage of liver cirrhosis, SIBO is related to the occurrence and development of BT, SBP, and endotoxemia. In liver cirrhosis, SIBO is one of the main factors that promote BT, and the occurrence of BT in mesenteric lymph nodes is usually related to SIBO in experimental models (51). However, BT does not occur in as many as half of SIBO’s cirrhotic models, so it seems that SIBO is permissible, but not enough in itself to cause BT to occur. Therefore, other factors, most likely, the decline in local immunity may play the most important role in inducing BT.

SIBO in liver cirrhosis is partly due to the movement of the small intestine and the decrease of the time passing through the intestine (52). The possible catalytic effect of proton pump inhibitors on SIBO and SBP has recently been questioned in many patients with liver cirrhosis. Nevertheless, it has been found that gastric acid deficiency has been observed in patients with liver cirrhosis without acid inhibitor used, resulting in an increase in pH in the small intestine, which promotes the production of SIBO (42).

It can be seen that the changes in the number and species of intestinal bacteria are related to liver cirrhosis, and maintaining the stability of intestinal bacteria plays an important role in controlling the occurrence and development of liver cirrhosis (53).

Bile acid is a new player to regulate intestinal flora. Although intestinal microbial ecological disorders and some intestinal flora may cause or aggravate liver cirrhosis, some reports in recent literature have shown that intestinal flora and its metabolites may have protective effects on the liver (54, 55). It has been recognized that both primary (hepatocytes derived) and secondary (microbial modified) bile acid (BA) act as signaling molecules in the human body. BA modulates its synthesis and regulation of key metabolic pathways and inflammatory responses by activating Farnesoid X receptor (FXR) (preferred primary BAs) and G protein-coupled receptors. Although the synthesis of BA is regulated by primary BA metabolism, the composition of intestinal flora is regulated by secondary BA. Therefore, the composition of BA, intestinal flora, and the delicate balance of living FXR activation has a profound effect on liver metabolism, anti-inflammation, and hepatoprotective process. For this reason, FXR continues to be explored as a potential treatment for liver cirrhosis in animal models and clinical trials. The above findings provide evidence for the protective effect of intestinal flora and its metabolites in the pathobiology of liver cirrhosis and disease (53, 56).

## Intestinal Flora Alteration Is Associated With Liver Cirrhosis Complications

The biggest challenge for cirrhosis is developing complications. The novel strategy to revert, or even slow down the progression is desperately needed in the clinic. With the progressive development of cirrhosis from compensated stage to decompensated stage, complications of liver cirrhosis are going to occur and are most likely also related to the more severe alteration of intestinal flora, including lower levers of Firmicutes and higher levers of *Bacteroidetes* (57). The gut flora plays a role in the development of infections and the pathogenesis of hepatic encephalopathy. Hepatic encephalopathy is characterized mainly by hyperammonemia. The increase of ammonia content in patients with hepatic encephalopathy may be due to the increase of ammonia production caused by liver dysfunction and intestinal bacterial disorder. The regulation of intestinal flora aims at reducing the number of ammonia-producing bacteria in the intestinal tract and reducing the production of ammonia, thus providing a new treatment for hepatic encephalopathy (57).

Spontaneous bacterial peritonitis is another complication of liver cirrhosis. It occurs due to the migration of intestinal bacteria to the abdominal cavity. Due to the combined effects of increased intestinal permeability and bacterial overgrowth, the risk increases with the progress of liver cirrhosis. As hepatic Encephalopathy, the standard treatment of spontaneous bacterial peritonitis also includes antibacterial therapy. When the intestinal flora is adjusted, the occurrence of spontaneous bacterial peritonitis may be reduced with decreasing in BT (58).

Bacillosis with liver cirrhosis is mainly caused by excessive growth of intestinal bacteria, immune dysfunction, and decreased bactericidal activity of phagocytes. Bacterial translocation leads to the damage of local or systemic immune defense mechanisms and plays an important role in the development of liver cirrhosis (59). Bacterial products lead to monocytes, lymphocytes activation, elevated serum TNF-α levels, inflammatory cytokines, and nitric oxide (NO) activation (60). The activation and elevated serum levels of NO lead to systemic vascular dilatation, increased cardiac output, decreased mean arterial pressure, and lead to complications of liver Cirrhosis, such as varicose veins, ascites, and hepatorenal syndrome (59, 61).

## Development of Novel Treatment for Cirrhosis by Targeting Microbiota and TLRs Against Cirrhosis

It is still a big challenge for the treatment of cirrhosis, and no cure medicines are available currently. The case with a clear diagnosis of cirrhosis by identifying liver pseudolobule is almost impossible to reverse the fibrosis to normal liver and the process of liver from compensating to decompensate and loss the function of liver almost cannot avoid eventually. The novel strategy/treatment is desperately needed in the clinic. Considering the gut dysbiosis is associated with the development of CLD, and then correcting the dysbiosis by modulating the gut microbiota may alter the course of the disease. Methods of modulating the gut microbiota include dietary modifications, antibiotic use, probiotics, prebiotics, and fecal microbial transplantation (FMT).

### Targeting Microbiome

The progress of liver cirrhosis can be slowed down by regulating intestinal flora (56). The main focus of the prevention of bacterial infections is the use of antibiotics prophylactically. Enterobacteriaceae and Streptococcus non-enterococci are the most common pathogenic microorganisms in liver cirrhosis, and the use of antibiotics is mainly aimed at these bacteria. Intestinal bacterial overgrowth in patients with liver cirrhosis is considered to be that intestinal is the most common site of bacterial translocation (62). Rifaximin is a broad-spectrum antibiotic which eliminates intestinal microorganisms non-selectively. Rifaximin can also directly affect bacterial function by weakening the translocation ability of intestinal flora (63). The activity of rifaximin is specific in the intestinal tract, and the experiment shows that it is not absorbed into the whole-body circulation, which reduces the toxicity or side effects of rifaximin in the whole body. Rifaximin has also been shown to have a remission effect on hepatic encephalopathy in patients with liver cirrhosis and has a beneficial trend in the control of infection and bleeding rate of varicose veins (64).

Another approach to modulate microbiota is utilizing lactulose, an approved laxative. The analysis of the therapeutic effect of lactulose in the study of hepatic encephalopathy showed that the withdrawal of lactulose led to cognitive deterioration, the decrease of bacterial content in feces, and the increase of glutamic acid and glutamine in the brain (65, 66). Therefore, it seems that the regulation of intestinal flora imbalance such as by rifaximin and lactulose is a promising strategy in the treatment of cirrhosis and hepatic encephalopathy.

Lactobacillus is protective against intestinal mucosa by lowering intestinal pH. They can prevent the establishment of pathogenic species and regulate the immune response and maintain the steady-state of intestinal flora. Improve overall intestinal function (67). In the study of the liver cirrhosis model, the application of lactobacteria has been shown to effectively reduce bacterial translocation and decrease the level of serum alanine aminotransferase, thus reducing the progress of cirrhosis (68).

Fecal microbial transplantation (FMT), as a mature treatment for refractory clostridium infection (69), and as a potential solution to reverse gut dysbiosis and its downstream complications in cirrhosis. A recent study showed that FMT can reduce mouse steatohepatitis by reducing pro-inflammatory cytokines in the liver and endotoxemia (70). In theory, FMT may also influence the disease’s trajectory and may alter the pathophysiology of the disease by reducing inflammation and fibrosis in the liver.

### Targeting TLRs

It appears that modulating microbiome can be utilized as an effective treatment against cirrhosis, and targeting the host receptors for these microbiomes may work too. Previous studies have reported targeted TLR therapy for liver disease. In an experimental model of chronic liver fibrosis, several toll-like receptors (TLRs) are needed to make mice sensitive to liver fibrosis. It was speculated that the ligands of TLR were bacterial products from the intestinal microbiome, and TLR knockout mice had resistance to liver inflammation and fibrosis (71). The intestinal microbiota plays an important role in the pathogenesis of both nonalcoholic fatty liver disease (NAFLD) and hepatocellular carcinoma (HCC). Moreover, TLR4 expression, alterations of bile acid metabolism, inflammatory cytokines released, may promote NAFLD-associated HCC (72). Therefore, potential targets for the prevention of HCC from NAFLD may include the composition of the microbiota, metabolites, and inhibition of TLRs. The relationship of intestinal microbiota with fibrosis development by identifying fibronectin as a TLR4 dependent mediator of the matrix and vascular changes that characterize cirrhosis (73). Intestinal sterilization confined to the late stage of HCC can reduce the occurrence of HCC, suggesting that the intestinal microbiota and TLR4 are therapeutic targets for HCC prevention in advanced liver disease (74).

However, many other approaches targeting TLRs to treat liver cirrhosis. Many TLR subtypes have been found in humans and are associated with oxidative stress (75). Oxidative stress reflects the imbalance between the production of reactive oxygen species (ROS) and the scavenging capacity of the antioxidant system. ROS leads to the progression of end-stage liver disease finally, and crosstalk between TLR and NADPH oxidase (NOX) is associated with fibrogenesis. The inhibitory effect of TLR-mediated oxidative stress in alleviating liver fibrosis has been demonstrated by a variety of natural drugs. Interestingly, the activity of natural derivatives was mainly inhibition of TLR4.

In preclinical studies, many natural drugs inhibit TLR4 or related signaling pathways to reduce oxidative stress, liver inflammation, and fibrosis (76). Besides, several compounds affecting TLR, namely curcumin, quercetin, probiotics, and have been found to have clinical effects on liver fibrosis-related diseases. Unfortunately, the link between the therapeutic efficacy of these compounds and TLR-related pathways has not been systematically tested in humans (77–79). The related inhibition of TGF- β1/Smad2 and TLR4/NF-κB p50 pathways with the prevention of liver inflammation and fibrosis emphasizes its potential as a therapeutic strategy for liver fibrosis (80).

Both probiotics and angiotensin-II type 1 receptor blocker (ARB) could inhibit hepatic fibrosis, accompanied by activation of hepatic stellate cells and inhibition of liver-specific transforming growth factor-β and TLR4 expression (81). Besides, ARB can improve hepatic fibrosis and inhibit the TLR4 signal through the angiotensin-II-mediated LPS-TLR4 signal (82).

## Concluding Remarks

The homeostasis of intestinal flora plays an important role in the human body and can be involved in the regulation of various functions of the human body. The homeostasis of intestinal flora has a protective effect on the liver. The imbalance of intestinal flora will be recognized by innate immune receptors such as Toll-like receptors. A chronic inflammatory response will destroy the liver, and will gradually aggravate liver cirrhosis, and promote the occurrence and development of complications of liver cirrhosis. Existing clinical evidence shows that drug treatment of intestinal flora disorders can slow down the progress of liver cirrhosis. With the research of intestinal flora affecting the development and progress of liver cirrhosis, it provides a direction for clinical treatment. The further study of flora composition and more human research will further enrich the clinical treatment and development and provide more treatment for delaying the progress of liver cirrhosis.

## Author Contributions

YF and YL wrote the manuscript. YC, JL, LC, and DZ wrote and critically revised the manuscript. All authors contributed to the article and approved the submitted version.

## Conflict of Interest

The authors declare that the research was conducted in the absence of any commercial or financial relationships that could be construed as a potential conflict of interest.
